# Upfront Aspirin-Free Antiplatelet Monotherapy After Percutaneous Coronary Intervention: A Systematic Review and Meta-Analysis of Safety and Efficacy^☆^

**DOI:** 10.1016/j.ihj.2026.04.002

**Published:** 2026-04-03

**Authors:** Kunal Mahajan, Deep Dutta, A.B.M. Kamrul-Hasan, Akshyaya Pradhan, Shekhar Vohra, Nitin Mahajan

**Affiliations:** a Department of Cardiology, Himachal Heart Institute, Mandi, Himachal Pradesh, India; b Department of Endocrinology, Center for Endocrinology Diabetes Arthritis & Rheumatism (CEDAR) Superspeciality Healthcare, Dwarka, New Delhi, India; c Department of Endocrinology, Mymensingh Medical College, Mymensingh, Bangladesh; d Department of Cardiology, King George Medical College, Lucknow, Uttar Pradesh, India; e Mount Sinai Morningside, Icahn School of Medicine at Mount Sinai, New York, USA; f Translational Research and Data Science Consultant, USA

**Keywords:** Aspirin, Dual antiplatelet therapy, Single antiplatelet therapy, Percutaneous coronary intervention

## Abstract

**Background:**

Evidence comparing non-aspirin single antiplatelet therapy (SAPT) with aspirin-containing dual antiplatelet therapy (DAPT) after percutaneous coronary intervention (PCI) is limited. We performed a systematic review and meta-analysis to evaluate the safety and efficacy of non-aspirin SAPT with a P2Y12​ inhibitor started at or within one week of PCI.

**Methods:**

Electronic databases were searched for studies evaluating non-aspirin SAPT after PCI. Primary outcomes were bleeding defined by Bleeding Academic Research Consortium (BARC) criteria [BARC 1–5 (any bleeding) and BARC 3–5 (major bleeding)], all-cause mortality (ACM), and cardiovascular mortality. Secondary outcomes were stroke, myocardial infarction (MI), need for revascularization, and stent thrombosis (STS).

**Results:**

Seven studies (2 randomized controlled trials and 5 observational studies) including 5468 patients on non-aspirin SAPT were analyzed. In the single-arm meta-analysis of non-aspirin SAPT, pooled prevalence was 5% (95% CI 3–11; I^2^ = 92%) for any BARC 1–5 bleeding, 3% (95% CI 1–7; I^2^ = 92.5%) for major BARC 3–5 bleeding, 2% (95% CI 1–3; I^2^ = 65.4%) for ACM, 2% (95% CI 2–3; I^2^ = 31%) for cardiovascular mortality, 1% (95% CI 1–1; I^2^ = 0%) for STS, 1% (95% CI 0–1; I^2^ = 40.1%) for stroke, 2% (95% CI 1–3; I^2^ = 66.6%) for MI, and 2% (95% CI 1–4; I^2^ = 75.9%) for revascularization. In pairwise analyses of the two trials, non-aspirin SAPT versus aspirin-based DAPT showed similar risks of all-cause mortality, cardiovascular mortality, bleeding, and stroke but higher risks of MI (odds ratio [OR]1.41; 95% CI 1.01–1.97; *P* = 0.05; I^2^ = 0%) and revascularization (OR1.73; 95% CI 1.18–2.52; *P* = 0.005; I^2^ = 0%).

**Conclusion:**

Upfront aspirin-free SAPT after PCI was associated with increased risks of MI and revascularization without a reduction in bleeding compared with aspirin-based DAPT.

## Graphical abstract

**Figure:**
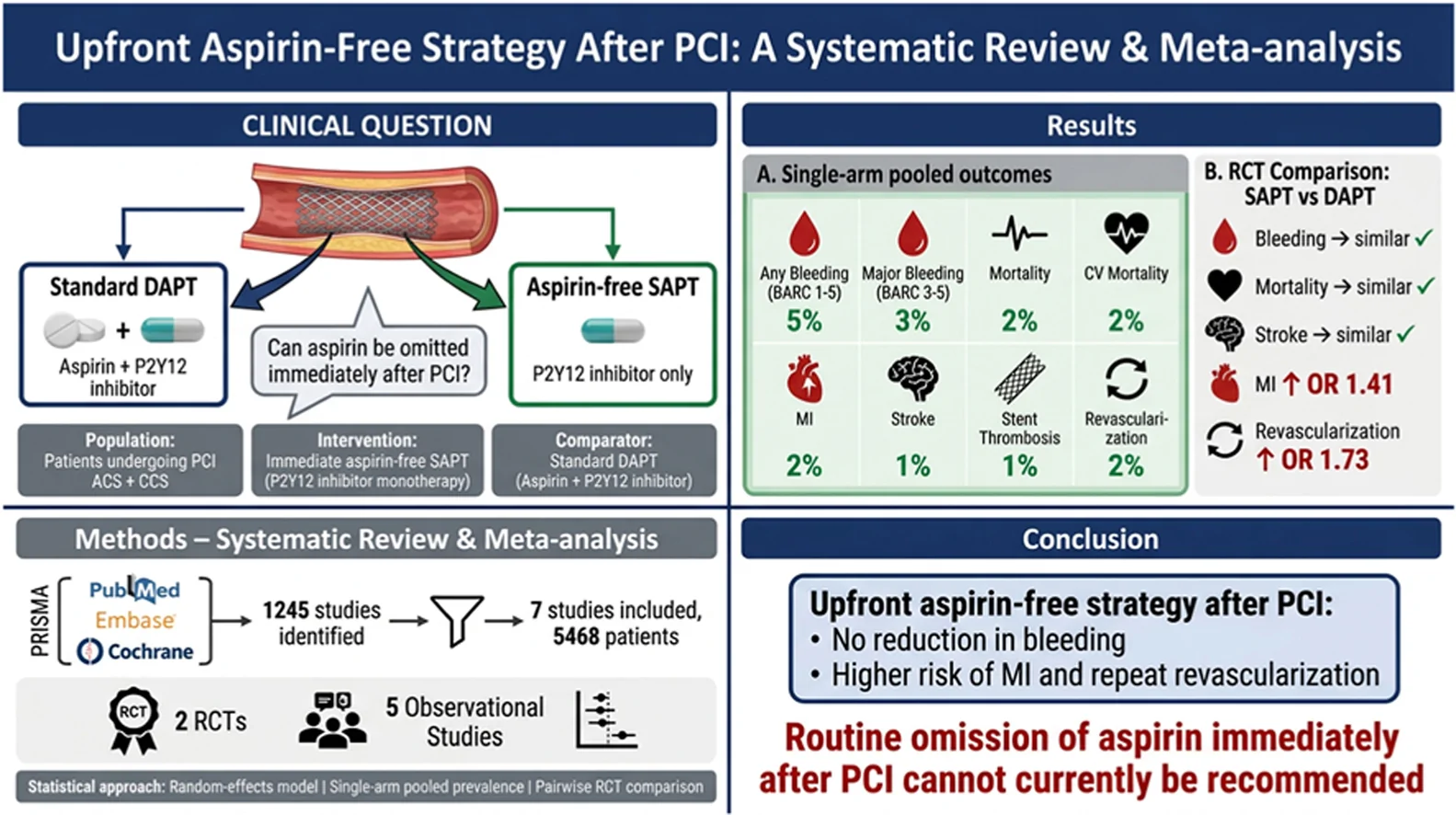

## 1 Introduction

Dual antiplatelet therapy (DAPT) with aspirin and a P2Y12 inhibitor has been the foundation of secondary prevention following percutaneous coronary intervention (PCI) for decades.^1^ Although this strategy is highly effective in reducing ischemic complications, such as stent thrombosis and recurrent myocardial infarction (MI), it inevitably increases the risk of bleeding, a complication that carries its own adverse prognostic implications.^2^ In the contemporary PCI era, characterized by thinner-strut drug-eluting stents, improved implantation techniques, and the advent of potent and consistent P2Y12 inhibitors such as ticagrelor and prasugrel, there is growing momentum to reconsider the necessity of aspirin in dual therapy.^3^

Recent randomized controlled trials (RCTs) have demonstrated that early discontinuation of aspirin after 1–3 months of DAPT, followed by P2Y12 inhibitor monotherapy, can meaningfully lower bleeding rates without increasing the risk of ischemic events.^4^ However, the early post-PCI phase remains a period of heightened vulnerability, when both thrombotic and hemorrhagic risks coexist.^5^ Therefore, the question arises: could the complete omission of aspirin from the outset, immediately after PCI, offer a safer yet equally effective alternative? This “aspirin-free” approach challenges long-standing conventions and seeks to simplify therapy while aligning antithrombotic intensity with contemporary procedural safety.

Emerging evidence from both randomized and exploratory studies has begun to address this possibility 6, 7, 8, 9, 10, 11, 12. The STOPDAPT-3 trial (Short and Optimal Duration of Dual Antiplatelet Therapy-3) which involved more than 5900 patients, reported that prasugrel monotherapy was non-inferior to DAPT for cardiovascular events at one month, albeit with a signal for higher stent thrombosis.^6^ Complementary data from smaller hypothesis-generating studies, such as OPTICA (Optical Coherence Tomography-Guided PCI with Single-Antiplatelet Therapy) study, MACT (Mono Antiplatelet and Colchicine Therapy) study, and the ASET (Acetyl Salicylic Elimination Trial) studies in Japan and Brazil, have demonstrated that early or immediate P2Y12 monotherapy is feasible and associated with reassuring safety in carefully selected low-risk patients 7, 8, 9, 10, 11, 12. A recent review integrating these trials suggested that while aspirin-free therapy appears safe and practical, its long-term ischemic equivalence to conventional DAPT remains uncertain and warrants comprehensive evaluation.^13^

The recently published NEO-MINDSET trial (Percutaneous Coronary Intervention Followed by Monotherapy Instead of Dual Antiplatelet Therapy in the Setting of Acute Coronary Syndromes) trial evaluated potent P2Y12 inhibitor monotherapy initiated within days of PCI in patients with acute coronary syndrome (ACS) and showed a marked reduction in bleeding, although it narrowly missed statistical non-inferiority for ischemic outcomes compared with standard DAPT.^14^ It must be realized that a holistic quantitative analysis of all the available data comparing the safety and efficacy outcomes of aspirin-free single antiplatelet therapy (SAPT) vs. aspirin-containing DAPT is still not available. In this context, we conducted a systematic review and meta-analysis (SRM) to quantitatively analyze all available RCT and observational evidence examining aspirin-free P2Y12 inhibitor monotherapy (SAPT) initiated at the time of PCI or within the first week after PCI. By pooling data across diverse populations—spanning ACS and chronic coronary syndromes (CCS)—and different P2Y12 inhibitory agents, this study aims to provide a clearer understanding of whether omitting aspirin altogether can maintain ischemic protection while mitigating bleeding, particularly in the vulnerable early post-procedural period.

## 2 Methods

### 2.1 Ethical compliance

This SRM was conducted according to the procedures outlined in the Cochrane Handbook for Systematic Reviews of Interventions and reported according to the Preferred Reporting Items for Systematic Reviews and Meta-Analyses (PRISMA) checklist.^15^ The SRM is registered with PROSPERO (CRD420251162398), and the protocol summary can be accessed online.

### 2.2 Search strategy

We conducted an extensive literature search across databases including PubMed, Embase, and Cochrane Library. All studies published up to 31st October 2025 were examined. The search strategy utilized combinations of key terms such as “aspirin”, “clopidogrel”, “prasugrel”, “ticagrelor”, “dual antiplatelet therapy”, “aspirin-free”, “coronary artery disease”, “percutaneous coronary intervention”, “angioplasty” and “acute coronary syndrome” using appropriate Boolean search operators “AND” and “OR” (Detailed search strategy is provided in supplementary material). The search terms were implemented in the titles and abstracts of the documents. The goal was to find published and unpublished full-length journal articles and abstracts for the conference proceedings in English. Additionally, the search entailed examining references in the published articles obtained for this study and in relevant journals.

### 2.3 Eligibility criteria

The PICOS criteria were utilized to screen and select the studies. The population (P) included people living with coronary artery disease who have undergone percutaneous coronary intervention (PCI). The intervention (I) consisted of the use of aspirin-free P2Y12 monotherapy with/without <5 days of DAPT post-PCI along with standard of care. The control (C) group (if available) received aspirin-based DAPT along with the standard of care. In addition, studies without a control group were considered for single-arm and proportion meta-analysis. The outcomes (O) focused on all-cause mortality, cardiovascular mortality, Bleeding Academic Research Consortium (BARC) bleeding scale, stroke, myocardial infarction, need for revascularization, stent thrombosis, and any other side effects. The study type (S) comprised RCTs, cohort studies, case-control studies, and case series.

Cross-sectional studies, case reports, reviews, expert opinions, editorials, letters to the editor, and duplicate reports were excluded from the analysis. Duplicates were removed before screening articles by title and abstract, followed by full-text screening to confirm eligibility.

### 2.4 Study outcomes

The primary outcomes analyzed were BARC 1-5 (any bleeding), BARC 3-5 (major bleeding), all-cause mortality, and cardiovascular mortality. Secondary outcomes included the occurrence of stroke, myocardial infarction, need for revascularization, stent thrombosis, and any other side effects.

### 2.5 Study selection

Two reviewers independently screened the titles and abstracts of the identified studies. They reviewed the full text to determine if a study could not be excluded based solely on the title and abstract. Any disagreements concerning the study's eligibility were resolved by consulting a third author.

### 2.6 Data extraction

Two review authors independently extracted data using standardized forms for data extraction. When multiple publications from a single study group were identified, the results were consolidated, and relevant data from each article were included in the analyses. The following data were extracted from all eligible studies and included in the review: first author, year of publication, the country where the study was conducted, study design, major inclusion and exclusion criteria, sample size, treatment received, and the outcomes mentioned above. Any disagreements were resolved by consensus.

### 2.7 Data synthesis and statistical analysis

The extracted data were analyzed using the “meta” and “metafor” packages in RStudio for R Statistics programming (Version 2025.4.5.0).^16^ Continuous variables were presented as mean/standard deviation, whereas categorical variables were presented as frequency/proportion. The outcomes were measured as mean differences (MD) for the continuous variables and odds ratios (OR) or risk ratios (RR) for the categorical variables, along with 95% confidence intervals (CI). The random-effects model was chosen to address the anticipated heterogeneity resulting from population characteristics in the included studies. The inverse variance statistical method was applied for all instances. Details of the meta-analysis included using the restricted maximum-likelihood estimator for tau^2^, the Q-profile method for the confidence interval of tau^2^ and tau. Calculation of I^2^ was based on Q statistics using untransformed (raw) means. Forest plots were generated with the same software. Forest plots were generated for visualization in RStudio. The Review Manager online software (RevMan Web) (Cochrane Collaboration UK, 2025) was used to compare the primary and secondary outcomes between the intervention and control groups for the RCTs. A *p*-value <0.05 was considered statistically significant.

### 2.8 Assessment of heterogeneity and publication bias

The assessment of heterogeneity was initially performed by examining forest plots. Subsequently, Chi-squared tests were conducted using N-1 degrees of freedom and a significance level of 0.05 to assess statistical significance. The I^2^ test was also used in the subsequent analysis. Thresholds for I^2^ values were defined as follows: 25% for low heterogeneity, 50% for moderate heterogeneity, and 75% for high heterogeneity.^17^

### 2.9 Assessment of the quality of the included studies

Two authors independently assessed the risk of bias (RoB). The Cochrane risk-of-bias tool for randomized trials version 2 (RoB2) was used to determine the RoB of the RCTs, whereas the Risk of Bias In Non-randomized Studies of Interventions version 2 (ROBINS-I V2) was used for non-randomized intervention trials and retrospective cohort studies.^18^^,^^19^ In cases of discrepancies, the fifth and sixth authors served as arbitrators to achieve consensus. The Risk-of-bias VISualization (robvis) web app was used to create risk-of-bias plots.^20^, ^21^

## 3 Results

An initial search revealed 1245 articles. 145 duplicates and many other articles that did not fulfil inclusion criteria were removed. Finally, data from 7 studies (5468 patients who received non-aspirin SAPT), which fulfilled all inclusion and exclusion criteria, were analyzed in this SRM 6, 7, 8, 9, 10, 11, 12^,^^14^ (Fig. 1). This includes 5 single-arm observational studies 7, 8, 9, 10, 11, 12 and 2 RCTs (STOPDAPT-3 trial and NEO-MINDSET trial).^6^^,^^14^ Van der Sangen et al published 2 papers from the same cohort of patients (OPTICA study)^7^^,^^8^; in our SRM, they were analyzed together. The OPTICA, MACT, and ASET were single-arm studies evaluating the impact of aspirin-free SAPT vs. aspirin-containing DAPT on cardiovascular outcomes.^8^^,^^9^^,^^12^ The study by Muramatsu et al was a single-arm study that evaluated prasugrel SAPT after PCI with a biodegradable-polymer platinum-chromium everolimus-eluting stent for Japanese patients with CCS (ASET-Japan Study).^10^ The study by Kogame et al was a single-arm study that evaluated prasugrel SAPT following PCI in patients with stable coronary artery disease (CAD) (ASET-pilot study).^11^ The details of the patients in the different studies analyzed in this SRM have been elaborated in Table 1. The differences in key features between the two RCTs are elaborated in Table 2.

**Figure-1:**
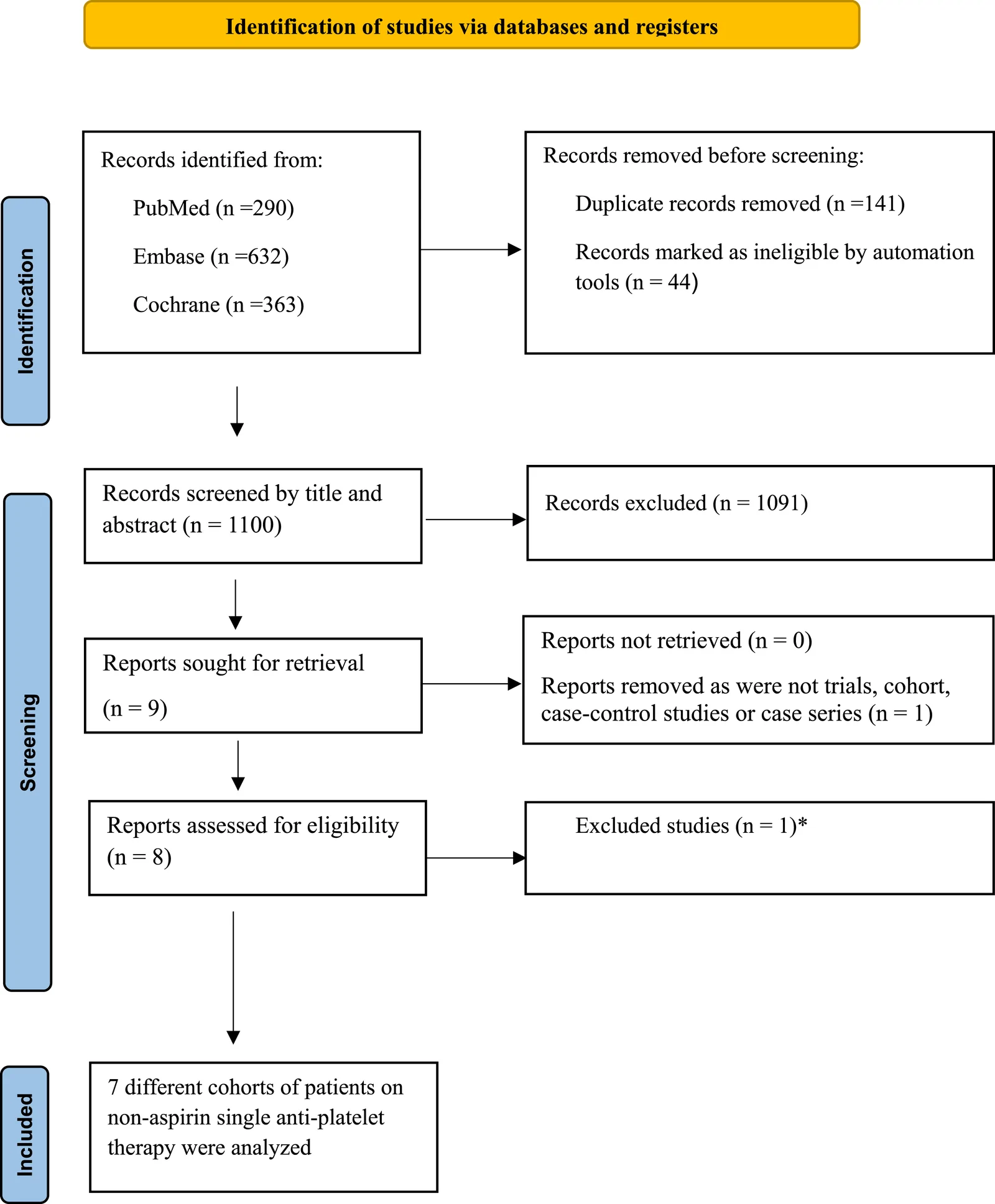
Flowchart elaborating on study retrieval and inclusion in this systematic review ∗Van der Sangen et al published 2 papers from the same cohort of patients (OPTICA study) (7,8); in our SRM they were analyzed together.

**Table 1:** Characteristics of patients analyzed in this systematic review and meta-analysis.

Study	Type of study	Population	Number of patients	P2Y12 inhibitor monotherapy group	DAPT group	Major outcome	Follow up (months after PCI)
Guimarães, 2025 (NEO-MINDSET trial)	RCT	ACS (STEMI:62.1%, NSTE-ACS:37.9%)	3410	P2Y12 inhibitor monotherapy (ticagrelor or prasugrel)	DAPT aspirin & potent P2Y12 inhibitor	Composite of death from any cause, MI, stroke, or urgent target-vessel revascularization & major or clinically relevant nonmajor bleeding (tested for superiority)	12 months
Miyashita, 2025 (ASET-Japan NSTE-ACS study)	Single arm observational study	ACS (100% NSTE-ACS)	101	prasugrel (3.75) mg once daily	None	The primary ischemic endpoint is a composite of cardiac death, spontaneous target vessel MI, or definite stent thrombosis; the primary bleeding endpoint is BARC Type 3 & 5 bleeding	12 months
Natsuaki 2023 (STOP DAPT 3 trial)	RCT	ACS (75 %) or non-ACS (25 %) Among ACS, 57% had STEMI and 43% had NSTE-ACS	5966	Prasugrel (3.75 mg once daily)	Aspirin (81–100 mg/d) & prasugrel (3.75 mg/d)	The coprimary end points were major bleeding (BARC 3 or 5) for superiority & cardiovascular events (a composite of cardiovascular death, MI, definite stent thrombosis, or ischemic stroke)	1 month
van der Sangen, 2023 (OPTICA study)	Single arm observational study	ACS (100% NSTE-ACS)	75	Ticagrelor (90 mg twice daily) or prasugrel (10 mg once daily)	None	Composite of all-cause mortality, MI, definite or probable stent thrombosis or stroke. The primary bleeding endpoint was BARC type 2, 3 or 5 bleeding.	12 months
Lee 2023 (MACT study)	Single arm observational study	ACS (STEMI: 46.5%, NSTE-ACS: 53.5%)	200	Ticagrelor (90 mg twice daily) or prasugrel (10 mg once daily)	None	The primary outcome was any stent thrombosis at 3 months.	3 months
Muramatsu, 2023 (ASET JAPAN study)	Single arm observational study	CCS with native de-novo coronary lesions & SYNTAX score <23	206	Low dose prasugrel (3.75 mg/day) monotherapy	None	The primary ischemic endpoint was a composite of cardiac death, spontaneous target-vessel MI, or definite stent thrombosis. The primary bleeding endpoint was BARC type 3 or 5.	3 months
Kogame, 2020 (ASET BRAZIL study)	Single arm observational study	CCS patients with a SYNTAX score <23	201	Prasugrel (3.75 mg once daily)	None	The primary ischemic endpoint was a composite of cardiac death, spontaneous target vessel MI, or definite stent thrombosis, & primary bleeding endpoint was BARC types 3 & 5 bleeding	3 months

BARC: Bleeding Academic Research Consortium; STOPDAPT-3: Short and Optimal Duration of Dual Antiplatelet Therapy-3; OPTICA: Optical Coherence Tomography-Guided PCI with Single-Antiplatelet Therapy; MACT: Mono Antiplatelet and Colchicine Therapy); ASET: Acetyl Salicylic Elimination Trial; NEO-MINDSET: Percutaneous Coronary Intervention Followed by Monotherapy Instead of Dual Antiplatelet Therapy in the Setting of Acute Coronary Syndromes; DAPT: dual anti-platelet therapy; SAPT: single anti-platelet therapy; MI: myocardial infarction; ACS: acute coronary syndrome; CCS: chronic coronary syndrome; NSTE: non-ST elevation; PCI: percutaneous coronary intervention.

**Table 2:** Comparison of NEO-MINDSET and STOPDAPT-3 trials.

Parameter	NEO-MINDSET Trial (NEJM 2025)	STOPDAPT-3 Trial (Circulation 2024)
Study Design	Multicenter, open-label, randomized trial comparing early P2Y12 inhibitor monotherapy vs standard DAPT in ACS patients.	Multicenter, open-label, randomized trial comparing immediate prasugrel monotherapy vs DAPT in ACS and HBR patients.
Study Sites and Enrolment	Brazil and 50 international sites; 3412 patients enrolled.	Japan; 72 hospitals; 5966 patients enrolled.
Population	Predominantly ACS (STEMI 62.1%, NSTEMI 30.5%, UA 7.4%).	ACS 75%, non-ACS with high bleeding risk 25%.
Timing of Randomization	Within 4 days of hospitalization, after successful PCI (post-procedure).	Before PCI.
Intervention (SAPT arm)	Ticagrelor 90 mg bid or Prasugrel 10 mg od monotherapy (no aspirin).	Prasugrel 3.75 mg od monotherapy (no aspirin).
Comparator (Control arm)	Standard DAPT with Aspirin 81–100 mg + P2Y12 inhibitor for 12 months.	DAPT with Aspirin 81–100 mg + Prasugrel 3.75 mg od for 1 month, followed by SAPT.
Primary Endpoint(s)	Dual primary endpoints (tested hierarchically): composite ischemic outcome (death, MI, stroke, stent thrombosis) and major bleeding (BARC 2–5) at 12 months.	Dual primary endpoints: composite cardiovascular event (death, MI, stroke, stent thrombosis, or TIMI major bleeding) and major bleeding (BARC 3–5) at 1 month.
Follow-up Duration	12 months.	1 month (primary); extended 12-month follow-up planned.
Ischemic Outcomes	7.0% vs 5.5% (risk difference +1.47 percentage points; noninferiority not met).	4.12% vs 3.69% (HR 1.12, 95% CI 0.84–1.50; noninferior).
Bleeding Outcomes	2.0% vs 4.9% (HR 0.40, 95% CI 0.26–0.59); lower bleeding with SAPT; superiority not formally tested (hierarchical design).	4.47% vs 4.71% (HR 0.95, 95% CI 0.74–1.24); no superiority for bleeding reduction.
Stent Thrombosis	12 vs 4 cases (numerically higher with SAPT).	0.58% vs 0.17% (HR ∼ 3.0–3.4); higher with SAPT.
Conclusion/Key Takeaway	Early P2Y12 inhibitor monotherapy after PCI in ACS reduced bleeding but narrowly missed noninferiority for ischemic events; optimal timing remains uncertain.	Immediate prasugrel monotherapy after PCI was noninferior for ischemic events but did not reduce bleeding and increased stent thrombosis.

ACS = acute coronary syndrome; ARC-HBR = Academic Research Consortium–High Bleeding Risk; BARC = Bleeding Academic Research Consortium; CCS = chronic coronary syndrome; CI = confidence interval; DAPT = dual antiplatelet therapy; HR = hazard ratio; MI = myocardial infarction; NI = noninferiority; PCI = percutaneous coronary intervention; RCT = randomized controlled trial; SAPT = single antiplatelet therapy; ST = stent thrombosis; STEMI = ST-segment elevation myocardial infarction; NSTEMI = non–ST-segment elevation myocardial infarction; TIMI = Thrombolysis in Myocardial Infarction; UA = unstable angina.

### 3.1 Risk of bias and quality assessment of the included studies

The risk of bias (ROB2)^21^ was rated as low among the RCTs included in this SRM. Details are provided in the supplementary Figure (SF) S1. The ROBINS-I risk of bias for the non-randomized studies analyzed in this SRM was low and has been detailed in SF S2. All the non-randomized studies had moderate risks of overall bias caused by confounding bias.

### 3.2 Primary outcomes

All seven studies report on BARC bleeding scores. The pooled prevalence (PP) of BARC 1-5 bleeding in patients on non-aspirin SAPT was 5% (95% CI: 0.03-0.11) having I^2^ of 92% (Fig. 2A). BARC 3-5 bleeding had a PP of 3% (95%CI: 1-7) having I^2^ 92.5% among patients on non-aspirin SAPT (Fig. 2B). The occurrence of all-cause mortality and cardiovascular mortality in patients on non-aspirin SAPT were 2% [95%CI: 1-3; I^2^ = 65.4%] and 2% (95%CI: 0.02-0.03; I^2^ = 31%) respectively (Fig. 2C and D).

**Figure-2:**
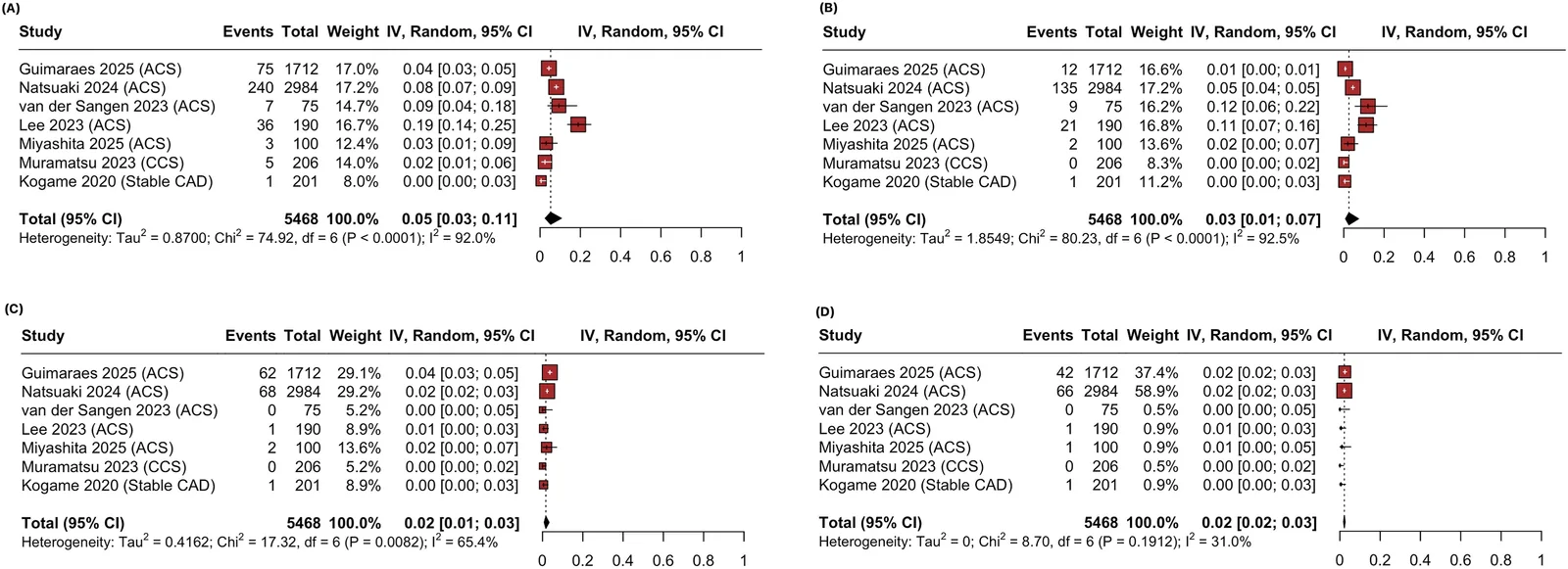
Pooled prevalence in patients on non-aspirin single antiplatelet therapy of (A): Bleeding Academic Research Consortium (BARC) 1-5 bleeding (any bleeding); (B): BARC 3-5 bleeding (major bleeding); (C): All-cause mortality; (D): Cardiovascular mortality.

### 3.3 Secondary outcomes

The prevalence of stent thrombosis, stroke, myocardial infarction, and need for revascularization in patients on non-aspirin SAPT was 1% [95%CI: 1-1; I^2^ = 0%; Fig. 3A], 1% [95%CI: 0-1; I^2^ = 40.1%; Fig. 3B], 2% [95%CI: 1-3; I^2^ = 66.6%; Fig. 3C], and 2% [95%CI: 1-4; I^2^ = 75.9%; Fig. 3D], respectively.

**Figure-3:**
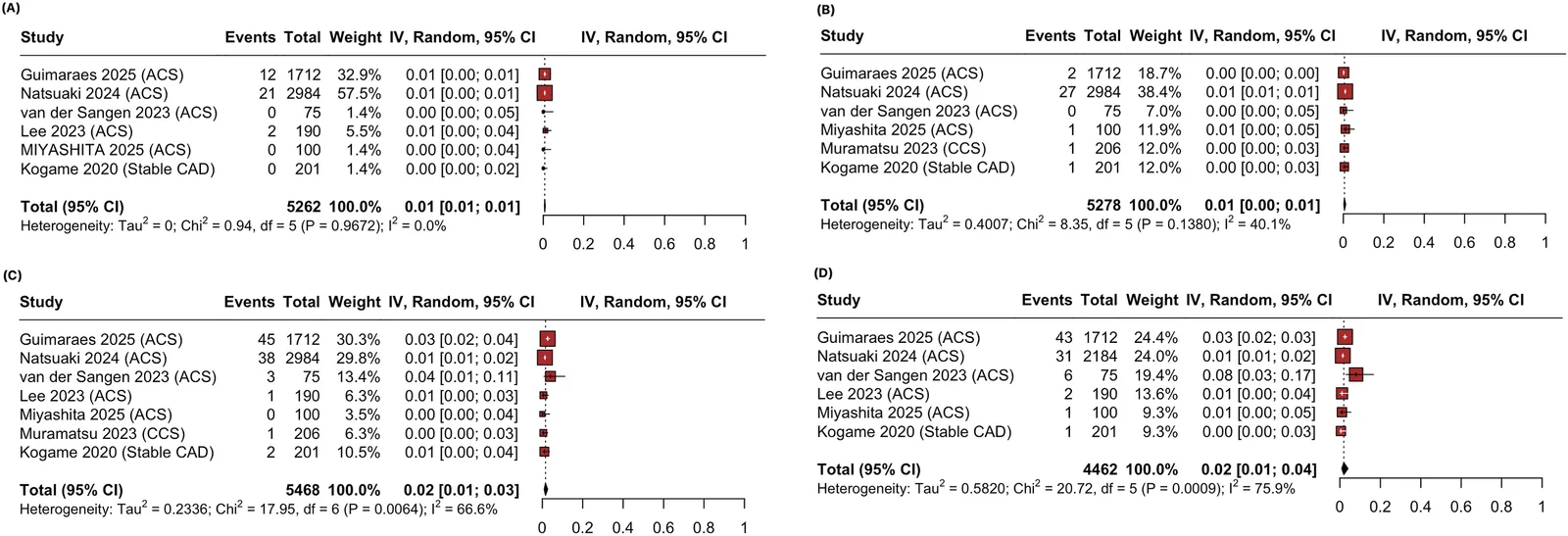
Pooled prevalence in patients on non-aspirin single antiplatelet therapy of (A):Stent thrombosis; (B): Stroke; (C): Myocardial infarction; (D): Revascularization.

### 3.4 Sub-analysis of data from RCTs

The risks of all-cause mortality [OR 1.15 [95%CI: 0.89-1.48; *P* = 0.28; I^2^ = 0%; Fig. 4A] and cardiovascular mortality [OR 1.14 [95%CI: 0.86-1.50; *P* = 0.37; I^2^ = 0%; Fig. 4B] in patients on non-aspirin SAPT were identical to those on aspirin-based DAPT.

**Figure-4:**
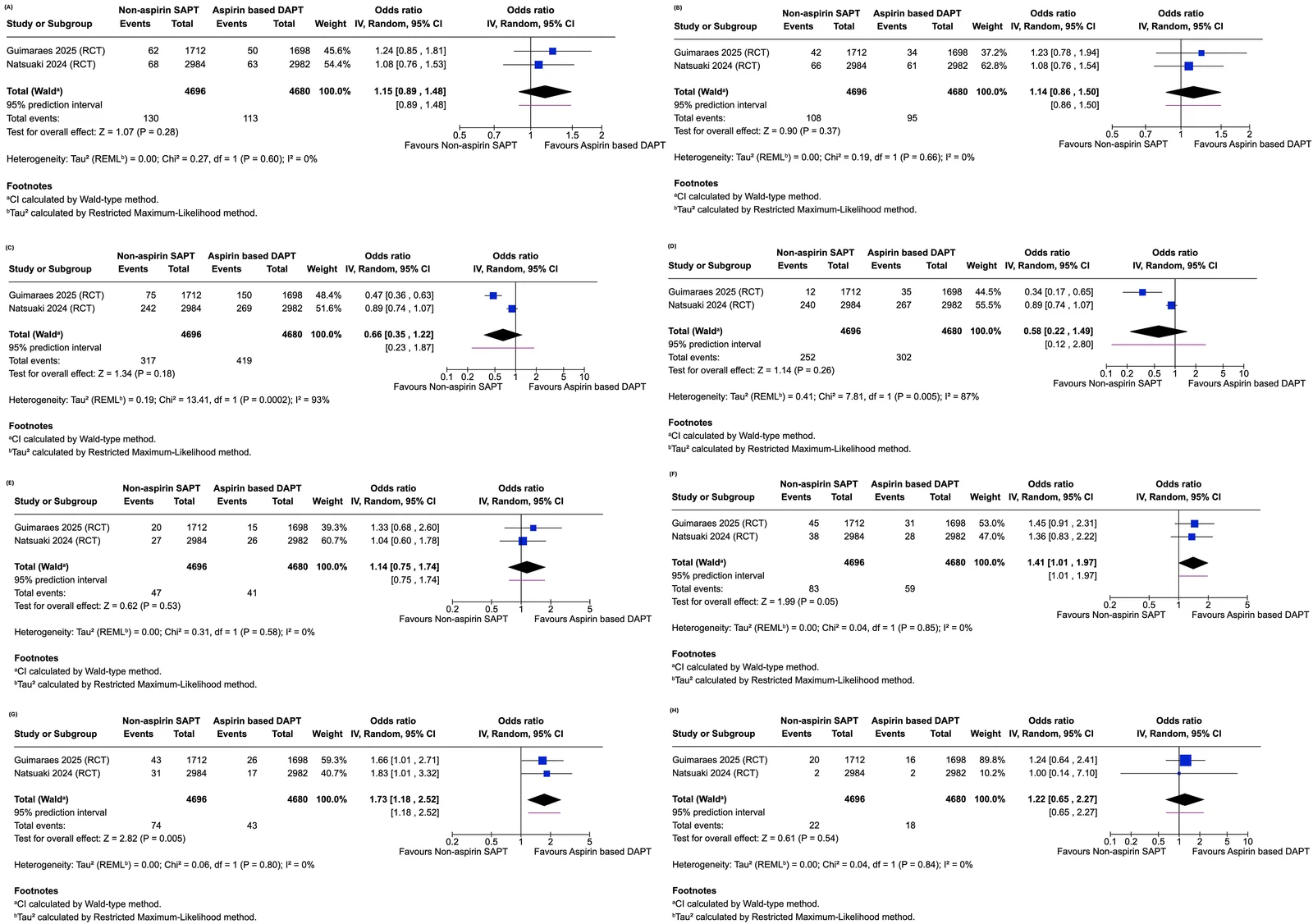
Impact of non-aspirin single antiplatelet therapy as compared to aspirin based dual anti-platelet therapy on risks of (A): All-cause mortality; (B): Cardiovascular mortality; (C): Bleeding Academic Research Consortium (BARC) 1-5 bleeding (any bleeding); (D): BARC 3-5 bleeding (major bleeding); (E): Stroke; (F): Myocardial infarction; (G): Revascularization; (H): Non-cardiovascular mortality.

The risks of overall bleeding (BARC bleeding 1-5) in patients on non-aspirin SAPT were similar to those on aspirin-based DAPT [OR 0.66 [95%CI: 0.35-1.22; *P* = 0.18; I^2^ = 93%; Fig. 4C]. Prediction interval analysis showed a significant increase in the CI [0.23-1.87] due to the presence of substantial data heterogeneity [Fig. 4C]. Similarly, the risks of major bleeding (BARC bleeding 3-5) in patients on non-aspirin SAPT were comparable to those on aspirin-based DAPT [OR 0.58 [95%CI: 0.22-1.49; *P* = 0.26; I^2^ = 87%; Fig. 4D]. Prediction interval analysis showed a significant increase in the CI [0.12-2.80] due to the presence of substantial data heterogeneity [Fig. 4D].

The risk of stroke in patients on non-aspirin SAPT was comparable to those on aspirin-based DAPT [OR 1.14 [95%CI: 0.75-1.74; *P* = 0.53; I^2^ = 0%; Fig. 4E]. The risk of MI in patients on non-aspirin SAPT was significantly higher compared to those on aspirin-based DAPT, which persisted on prediction interval analysis [OR 1.41 [95%CI: 1.01-1.97; *P* = 0.05; I^2^ = 0%; Fig. 4F]. The need for revascularization in patients on non-aspirin SAPT was significantly higher as compared to those on aspirin-based DAPT, which persisted on prediction interval analysis [OR 1.73 [95%CI: 1.18-2.52; *P* = 0.005; I^2^ = 0%; Fig. 4G]. The risk of non-cardiovascular mortality in patients on non-aspirin SAPT was similar to those on aspirin-based DAPT, which persisted on prediction interval analysis [OR 1.22 [95%CI: 0.65-2.27; *P* = 0.54; I^2^ = 0%; Fig. 4F].

## 4 Discussion

Current guidelines continue to recommend 12 months of DAPT with aspirin plus a potent P2Y12 inhibitor for patients with ACS after stent implantation, reflecting the persistently high early thrombotic risk after plaque rupture and PCI ^1^^,^^2^. Parallel to this standard, a growing body of evidence shows that withdrawing aspirin after 1–3 months of DAPT and continuing P2Y12 inhibitor monotherapy can reduce bleeding without a penalty in ischemic events compared with 12-month DAPT in selected patients.^4^^,^^13^ The present meta-analysis was designed to address a different and more provocative question: what happens when aspirin is omitted immediately or within the first week after PCI, and antithrombotic protection relies on SAPT with a potent P2Y12 inhibitor from the outset?

Our analysis was conducted from two complementary perspectives. First, we assessed event rates associated with an aspirin-free strategy by pooling the SAPT arms from two randomized controlled trials and several single-arm studies that included both acute and chronic coronary syndrome populations 6, 7, 8, 9, 10, 11, 12, 13, 14. This single-arm analysis provides a descriptive overview of safety, showing that P2Y12 inhibitor monotherapy without aspirin is feasible and generally well-tolerated, with low absolute rates of mortality, stroke, and major bleeding, but with some residual risk of myocardial infarction, stent thrombosis, and repeat revascularization. Collectively, these findings illustrate the expected safety profile when aspirin is omitted immediately or shortly after PCI, while also reflecting differences in patient type, choice of P2Y12 inhibitor, and procedural complexity across studies.

Second, and analytically distinct, we performed a comparative pooled analysis restricted to the two randomized trials that predominantly enrolled ACS patients—STOPDAPT-3 and NEO-MINDSET—to answer the clinical question that most directly informs ACS practice: how does immediate/very-early aspirin withdrawal compare with conventional DAPT when thrombotic risk is highest? In this ACS-focused comparison, aspirin-free SAPT did not reduce bleeding versus DAPT over the time frames studied and was associated with a higher risk of MI and unplanned revascularization, without a difference in mortality or stroke. These findings provide a pragmatic counterweight to the descriptive single-arm rates and emphasize that what looks feasible in mixed-risk cohorts may not translate into a net advantage in ACS when tested head-to-head against DAPT.

The divergent signals between descriptive and comparative views are explainable by timing, clinical phenotype, and pharmacologic intensity. STOPDAPT-3 randomized patients before PCI, tested low-dose prasugrel (3.75 mg/day) in a predominantly East Asian cohort, set 1-month coprimary endpoints, and found non-inferiority for cardiovascular events but no bleeding reduction and more stent thrombosis with SAPT versus DAPT.^6^ NEO-MINDSET randomized within 4 days after PCI in a global ACS cohort using standard-dose ticagrelor or prasugrel, observed substantially less clinically relevant bleeding with SAPT over 12 months, yet missed non-inferiority for the ischemic composite (Table 2).^14^

These trials suggest that immediate aspirin omission, during peak platelet turnover, thrombin generation, and endothelial injury, reduces the ischemic safety margin without substantially lowering bleeding risk. In contrast, prior trials and meta-analyses have shown that short early DAPT coverage for 1–3 months, followed by P2Y12 inhibitor monotherapy, restores the balance toward less bleeding while maintaining acceptable ischemic protection in most ACS patients, albeit with a slight trade-off in ischemic events 22, 23, 24. These temporal and biologic dynamics matter for clinical translation. Aspirin causes thromboxane A_2_ suppression on top of ADP-pathway inhibition; in the first days after PCI, residual thromboxane activity, pro-inflammatory signaling, and high platelet turnover can sustain thrombogenicity despite potent P2Y12 blockade. Therefore, removing aspirin too early can expose vulnerable non-culprit plaques and fresh strut surfaces to unopposed activation, particularly in ACS, complex PCI (long stent length, bifurcations), diabetes, or multivessel disease. As healing progresses and the thrombotic milieu cools, the bleeding liability of aspirin looms larger while its incremental ischemic benefit contracts—supporting phased de-escalation rather than blanket omission.^24^

Population and pharmacogenomic context further modulate effect size. STOPDAPT-3's low-dose prasugrel strategy reflects East Asian dosing norms and may deliver less platelet inhibition than Western regimens, potentially reducing the buffer against stent thrombosis under an aspirin-free plan.^6^ NEO-MINDSET used full-dose ticagrelor/prasugrel and still recorded a slight ischemic excess with SAPT despite less bleeding.^14^ These differences and high intravascular imaging use, stent platforms, and operator technique caution against one-size-fits-all extrapolation across geographies and practice environments.

What then should clinicians do? The combined evidence supports precision-guided antiplatelet therapy anchored to bleeding and ischemic phenotypes. Patients at high bleeding risk—advanced age, frailty, prior intracranial hemorrhage, anemia, chronic kidney disease, or concomitant oral anticoagulation—may reasonably be considered for early aspirin withdrawal with potent P2Y12 monotherapy once procedural success and haemostasis are secured, consistent with the spirit of short-DAPT data.^4^^,^^13^ Conversely, for ACS patients, especially those with complex PCI or high thrombotic burden, our ACS-restricted RCT analysis argues for at least a brief DAPT window before any de-escalation, in line with current ACS guidance favouring 12 months of DAPT as default, with individualized tailoring when bleeding risk dominates ^1^^,^^2^. In practice, integrating PRECISE-DAPT and ARC-HBR scoring with intraprocedural results and anatomy can operationalize this tailoring at the bedside ^25^^,^^26^.

Two additional observations deserve emphasis. First, the absence of a bleeding reduction in the ACS-focused RCT comparison does not negate the potential for later bleeding benefits with SAPT; many bleeds occur beyond the early time points used for the coprimary analyses (1-month in the STOPDAPT-3 trial, and 12-months in the NEO-MINDSET). Longer follow-up in diverse settings will be essential to clarify whether a delayed bleeding advantage emerges once periprocedural hazards recede. Second, the modest increase in MI and revascularization seen in the comparative ACS analysis should be viewed through an absolute-risk lens and weighed against patient-specific bleeding hazards rather than as a categorical indictment of SAPT strategies.

This study has limitations. The limited number of studies and their small sample sizes hinder definitive conclusions about the outcome estimates. The single-arm part of our analysis was crucial in demonstrating real-world SAPT event rates. However, it can be affected by selection bias and confounding. This is because operators might avoid using aspirin-free antiplatelet therapy in cases with a high risk of ischemic events. The ACS-focused comparative analysis is anchored on just two RCTs that differ in timing, dosing, and follow-up, limiting precision around subgroup effects. Follow-up durations were short in parts of the dataset, and very late thrombotic safety with immediate SAPT remains incompletely defined. Moderate to high heterogeneity for many outcome estimates limits the generalizability of the results. These caveats notwithstanding, the internal consistency of our two-tiered approach—descriptive rates under SAPT across ACS/CCS, then a focused RCT comparison in predominantly ACS—supports the central conclusion.

## 5 Conclusion

Our meta-analysis clarifies *when* and *for whom* an aspirin-free strategy may be appropriate after PCI. The single-arm analysis demonstrates that early P2Y12 monotherapy is feasible across mixed ACS and CCS populations with acceptable event rates. However, the pooled analysis of RCTs that predominantly included patients with ACS indicates that immediate aspirin omission provides no significant bleeding advantage and is associated with a modest increase in ischemic events compared with standard DAPT. The practical implications of our findings are consistent with current guideline recommendations, which endorse 12 months of DAPT as the default strategy for patients with ACS following PCI. Nevertheless, in individuals where the risk of bleeding outweighs the risk of ischemia and clinical stability has been achieved, a selective and appropriately timed withdrawal of aspirin in favour of potent P2Y12 inhibitor monotherapy may represent a reasonable, patient-centered approach. Although several recent trials and meta-analyses have reported favourable outcomes with de-escalation to monotherapy after 1–3 months of DAPT, the concept of an upfront aspirin-free strategy still requires further validation before it can be adopted in routine clinical practice. Future research incorporating genetic profiling, platelet function testing, and intravascular imaging to assess stent healing will be essential to refine the optimal timing and to identify those patient subsets who can safely discontinue aspirin early.

## Authors’ contribution

KM conceptualized the study. KM, DD, AP, SV, and NM conducted a literature search. DD and ABMKH entered data and analyzed it. AP, SV, and NM critically reviewed the manuscript for important intellectual content. All authors contributed equally to manuscript preparation. All authors take responsibility for all aspects of the reliability and freedom from bias of the data presented and their discussed interpretation.

## Funding

No funding has been received from any sources, including any pharmaceutical company.

## Declaration of competing interest

The authors declare that they have no known competing financial interests or personal relationships that could have appeared to influence the work reported in this paper.
