# Supplementary material for: Upfront Aspirin-Free Antiplatelet Monotherapy After Percutaneous Coronary Intervention: A Systematic Review and Meta-Analysis of Safety and Efficacy
Source: Indian Heart J. 2026 Apr 3;78(5):238–45. doi: 10.1016/j.ihj.2026.04.002 (PMC13751147; doi:10.1016/j.ihj.2026.04.002)
Supplement: Supplementary file 1 [file mmc1.docx]

**ONLINE ONLY SUPPLEMENTARY MATERIAL**

**Supplementary Figure-S1: Risk of bias (ROB2) of all the randomised controlled trials analyzed in this systematic review and meta-analysis**

**Supplementary Figure-S2: ROBINS-I risk of bias of non-randomised studies analyzed in this systematic review and meta-analysis**

Search Strategy used for PubMed/Embase/Cochrane

((("myocardial infarction" OR "acute coronary syndrome") AND ("ST segment elevation" OR "ST elevation" OR "ST elevated" OR "non ST")) OR ACS OR "unstable angina" OR "coronary stenting" OR "percutaneous coronary intervention" OR "PCI"))) AND ("P2Y12 monotherapy" OR "aspirin free" OR "single antiplatelet" OR "early withdrawal")
